# Supplementary material for: Resolving the full spectrum of human genome variation using Linked-Reads
Source: Genome Res. 2019 Apr;29(4):635–45. doi: 10.1101/gr.234443.118 (PMC6442396; doi:10.1101/gr.234443.118)
Supplement: Supplemental Material [file supp_gr.234443.118_Supplemental_longranger-2.2.zip › longranger-2.2.tar.bz2!/lr2.2-public/lib/stan/cmdstan/stan/doc/api/html/structstan_1_1gm_1_1constrained__param__names__visgen.html]

Stan: stan::gm::constrained\_param\_names\_visgen Struct Reference


|  |  |
| --- | --- |
|  | Stan  2.5.0  probability, sampling & optimization |


- Main Page
- Related Pages
- Namespaces
- Classes
- Files

- Class List
- Class Index
- Class Hierarchy
- Class Members

All Classes Namespaces Files Functions Variables Typedefs Enumerations Enumerator Friends Macros Pages

- stan
- gm
- constrained\_param\_names\_visgen

Public Member Functions |
List of all members

stan::gm::constrained\_param\_names\_visgen Struct Reference

`#include <generator.hpp>`

Inheritance diagram for stan::gm::constrained\_param\_names\_visgen:

|  |  |
| --- | --- |
| Public Member Functions | |
|  | constrained\_param\_names\_visgen (std::ostream &o) |
|  | |
| void | operator() (const nil &) const |
|  | |
| void | operator() (const int\_var\_decl &x) const |
|  | |
| void | operator() (const double\_var\_decl &x) const |
|  | |
| void | operator() (const vector\_var\_decl &x) const |
|  | |
| void | operator() (const row\_vector\_var\_decl &x) const |
|  | |
| void | operator() (const matrix\_var\_decl &x) const |
|  | |
| void | operator() (const unit\_vector\_var\_decl &x) const |
|  | |
| void | operator() (const simplex\_var\_decl &x) const |
|  | |
| void | operator() (const ordered\_var\_decl &x) const |
|  | |
| void | operator() (const positive\_ordered\_var\_decl &x) const |
|  | |
| void | operator() (const cholesky\_factor\_var\_decl &x) const |
|  | |
| void | operator() (const cholesky\_corr\_var\_decl &x) const |
|  | |
| void | operator() (const cov\_matrix\_var\_decl &x) const |
|  | |
| void | operator() (const corr\_matrix\_var\_decl &x) const |
|  | |
| void | generate\_param\_names\_array (const std::vector< expression > &matrix\_dims, const std::string &name, const std::vector< expression > &dims) const |
|  | |
| Public Member Functions inherited from stan::gm::visgen | |
|  | visgen (std::ostream &o) |
|  | |

|  |  |
| --- | --- |
| Additional Inherited Members | |
| Public Types inherited from stan::gm::visgen | |
| typedef void | result\_type |
|  | |
| Public Attributes inherited from stan::gm::visgen | |
| std::ostream & | o\_ |
|  | |

## Detailed Description

Definition at line 3196 of file generator.hpp.

## Constructor & Destructor Documentation

|  |  |  |  |  |  |
| --- | --- | --- | --- | --- | --- |
| stan::gm::constrained\_param\_names\_visgen::constrained\_param\_names\_visgen | ( | std::ostream & | *o* | ) |  |

inline

Definition at line 3197 of file generator.hpp.

## Member Function Documentation

|  |  |  |  |
| --- | --- | --- | --- |
| void stan::gm::constrained\_param\_names\_visgen::generate\_param\_names\_array | ( | const std::vector< expression > & | *matrix\_dims*, |
|  |  | const std::string & | *name*, |
|  |  | const std::vector< expression > & | *dims* |
|  | ) |  | const |

inline

Definition at line 3268 of file generator.hpp.

|  |  |  |  |  |  |
| --- | --- | --- | --- | --- | --- |
| void stan::gm::constrained\_param\_names\_visgen::operator() | ( | const nil & |  | ) | const |

inline

Definition at line 3200 of file generator.hpp.

|  |  |  |  |  |  |
| --- | --- | --- | --- | --- | --- |
| void stan::gm::constrained\_param\_names\_visgen::operator() | ( | const int\_var\_decl & | *x* | ) | const |

inline

Definition at line 3201 of file generator.hpp.

|  |  |  |  |  |  |
| --- | --- | --- | --- | --- | --- |
| void stan::gm::constrained\_param\_names\_visgen::operator() | ( | const double\_var\_decl & | *x* | ) | const |

inline

Definition at line 3204 of file generator.hpp.

|  |  |  |  |  |  |
| --- | --- | --- | --- | --- | --- |
| void stan::gm::constrained\_param\_names\_visgen::operator() | ( | const vector\_var\_decl & | *x* | ) | const |

inline

Definition at line 3207 of file generator.hpp.

|  |  |  |  |  |  |
| --- | --- | --- | --- | --- | --- |
| void stan::gm::constrained\_param\_names\_visgen::operator() | ( | const row\_vector\_var\_decl & | *x* | ) | const |

inline

Definition at line 3212 of file generator.hpp.

|  |  |  |  |  |  |
| --- | --- | --- | --- | --- | --- |
| void stan::gm::constrained\_param\_names\_visgen::operator() | ( | const matrix\_var\_decl & | *x* | ) | const |

inline

Definition at line 3217 of file generator.hpp.

|  |  |  |  |  |  |
| --- | --- | --- | --- | --- | --- |
| void stan::gm::constrained\_param\_names\_visgen::operator() | ( | const unit\_vector\_var\_decl & | *x* | ) | const |

inline

Definition at line 3223 of file generator.hpp.

|  |  |  |  |  |  |
| --- | --- | --- | --- | --- | --- |
| void stan::gm::constrained\_param\_names\_visgen::operator() | ( | const simplex\_var\_decl & | *x* | ) | const |

inline

Definition at line 3228 of file generator.hpp.

|  |  |  |  |  |  |
| --- | --- | --- | --- | --- | --- |
| void stan::gm::constrained\_param\_names\_visgen::operator() | ( | const ordered\_var\_decl & | *x* | ) | const |

inline

Definition at line 3233 of file generator.hpp.

|  |  |  |  |  |  |
| --- | --- | --- | --- | --- | --- |
| void stan::gm::constrained\_param\_names\_visgen::operator() | ( | const positive\_ordered\_var\_decl & | *x* | ) | const |

inline

Definition at line 3238 of file generator.hpp.

|  |  |  |  |  |  |
| --- | --- | --- | --- | --- | --- |
| void stan::gm::constrained\_param\_names\_visgen::operator() | ( | const cholesky\_factor\_var\_decl & | *x* | ) | const |

inline

Definition at line 3243 of file generator.hpp.

|  |  |  |  |  |  |
| --- | --- | --- | --- | --- | --- |
| void stan::gm::constrained\_param\_names\_visgen::operator() | ( | const cholesky\_corr\_var\_decl & | *x* | ) | const |

inline

Definition at line 3249 of file generator.hpp.

|  |  |  |  |  |  |
| --- | --- | --- | --- | --- | --- |
| void stan::gm::constrained\_param\_names\_visgen::operator() | ( | const cov\_matrix\_var\_decl & | *x* | ) | const |

inline

Definition at line 3255 of file generator.hpp.

|  |  |  |  |  |  |
| --- | --- | --- | --- | --- | --- |
| void stan::gm::constrained\_param\_names\_visgen::operator() | ( | const corr\_matrix\_var\_decl & | *x* | ) | const |

inline

Definition at line 3261 of file generator.hpp.

---

The documentation for this struct was generated from the following file:

- src/stan/gm/generator.hpp


---

[ Stan Home Page ]

*© 2011–2014,
Stan Development Team.*
